# Exploring measures of sustainability in the WHO Joint External Evaluation and the WOAH performance of veterinary services tools—A qualitative assessment of perceived usefulness and acceptability to one health and global health security experts

**DOI:** 10.1371/journal.pone.0343801

**Published:** 2026-03-04

**Authors:** Osman Ahmed Dar, Max Claron, Hassaan Zahid, Mishal Khan, Neil Spicer

**Affiliations:** 1 Global Public Health Directorate, United Kingdom Health Security Agency, London, United Kingdom; 2 Friendship France, Paris, France; 3 Medicins Sans Frontiers, Juba, South Sudan; 4 London School of Hygiene and Tropical Medicine, London, United Kingdom; Nottingham Trent University, UNITED KINGDOM OF GREAT BRITAIN AND NORTHERN IRELAND

## Abstract

**Introduction:**

Amidst global policy reforms including the 2024 amendments to the International Health Regulations (IHR) and the ongoing Pandemic Agreement negotiations, there is a renewed emphasis on sustainability, equity, and multisectoral coordination in global health security. However, the operationalization of sustainability in core assessment tools—such as the WHO Joint External Evaluation (JEE) and the WOAH Performance of Veterinary Services (PVS)—remains poorly defined and underdeveloped, particularly as they relate to low- and middle-income countries (LMICs).

**Methods:**

We conducted a qualitative study involving semi-structured interviews with 29 global experts across human, animal and environment health from both high and low/middle income countries affiliated with the One Health High-Level Expert Panel, the World Bank Pandemic Fund Technical Advisory Panel, and technical focal points from Quadripartite institutions. Using thematic analysis grounded in the Social Construction Framework and an adapted Schell et al. sustainability model, we explored how sustainability is conceptualized and measured across human, animal, and environmental health domains.

**Results:**

Participants critiqued existing tools for framing sustainability narrowly, including their focus on short-term processes and compliance, rather than long-term outcomes, equity, or resilience. Sectoral and contextual differences emerged: human health experts emphasized workforce and financing; animal health experts stressed economic and institutional continuity; environmental health experts highlighted ecosystem resilience and intergenerational equity. LMIC respondents underscored the impact of donor dependency, weak local ownership, and limited transition planning. Several determinants of sustainability—such as financing, governance, workforce retention, and community engagement—are already measured in existing reporting tools but are not explicitly or coherently framed as sustainability indicators.

**Discussion:**

We recommend that future iterations of JEE and PVS incorporate a clear definition of sustainability and explicitly integrate sustainability metrics, aligned with the revised IHR, the One Health Joint Plan of Action, relevant SDG targets and national planning cycles. Tools must also reflect sectoral and contextual nuances and integrate long-term monitoring frameworks that promote domestic accountability. In the field of One Health and GHS, strengthening the sustainability components of these tools is essential to build equitable and resilient health systems globally.

## Introduction

Global health security (GHS) encompasses the proactive and reactive measures taken to prevent, detect, and respond to infectious disease and other public health threats that transcend national borders [1]. Its modern origins can be traced back to the 19th-century International Sanitary Conferences (ISC), where the primary objective was to safeguard the health of populations in imperial centers from diseases perceived to originate in the colonies. These conferences sought standardized quarantine measures to protect trade and prevent the spread of diseases such as smallpox, cholera, plague, and yellow fever [2]. The ISCs laid the groundwork for the creation of the World Health Organization (WHO) and subsequent international health agreements and other institutions, embedding colonial attitudes and priorities into the fabric of global health governance. The International Health Regulations (IHR), first adopted in 1969 to address ISC-focused diseases were revised and expanded in 2005 to an all-hazards approach and represent a legally binding framework for GHS [3,4]. Despite their importance and a further revision in 2024, critiques persist regarding the IHR’s efficacy, enforceability and equity, particularly concerning the capacities of low- and middle-income countries (LMICs) to comply with and benefit from the regulations [5,6].

The COVID-19 pandemic exposed and exacerbated these systemic weaknesses, underscoring the need for a more robust, inclusive, and sustainable GHS architecture. In response, WHO Member States initiated negotiations in 2021 for a new Pandemic Agreement, yet deep divisions persist between high-income countries (HICs) and LMICs, on equitable access to vaccines, pathogen sharing, and intellectual property rights [7–9]. The parallel IHR revision process in 2024 has placed greater emphasis placed on incorporating One Health principles, sustainability, and equitable access to medical countermeasures and financing [4,6]. These developments mark a critical policy shift in global health governance: equity, multisectoral collaboration, and long-term resilience are now central pillars of health security.

The integration of sustainability into GHS discourse grew after the publication of the Brundtland Report in 1987, which defined sustainable development as that which “meets the needs of the present without compromising the ability of future generations to meet their own needs” [10]. That report kickstarted an international process to harmonize and unify efforts around three pillars of development – society (including health), economy and environment – culminating with world leaders committing in 2015 to achieving 17 Sustainable Development Goals (SDGs) by 2030 [11]. Sustainability in GHS implies the enduring ability of health systems to manage and recover from shocks, particularly in resource-constrained environments. However, achieving sustainability is increasingly jeopardized by a contraction in donor funding. Significant reductions by major donors such as the United Kingdom and the United States have led to programme closures, workforce reductions, and risks to disease control efforts in several LMICs [12–14]. UNAIDS has announced workforce cuts exceeding 50%, while WHO has warned that several countries may soon run out of HIV medicines [14–16]. Only 17% of assessable SDG targets remain on track for 2030 [17]. In this context, sustainability is both a strategic and ethical imperative for GHS.

The One Health approach offers a promising avenue to advance sustainability by recognizing that the health of humans, domestic and wild animals, plants, and the wider environment (including ecosystems) are closely linked and interdependent. The approach encourages multisectoral collaboration, transdisciplinarity and resource-sharing, and is predicated on principles of equity, inclusiveness and responsible stewardship [18–20]. Its application is particularly valuable in LMICs, where human health is often deeply interwoven with agricultural practices, livestock management, and environmental change. Moreover, One Health can enhance program efficiency in resource-limited settings by enabling integrated surveillance, shared infrastructure, and joint emergency response mechanisms [21–23]. In the current funding climate, such cross-sectoral integration can provide a buffer against systemic fragility and promote better health and well-being outcomes for humans, animals and ecosystems at lower cost.

Nevertheless, sustainability remains variably defined and inconsistently measured across One Health and GHS frameworks [24]. In One Health, sustainability is often understood in ecological and systems terms whereas in GHS it is more closely tied to institutional capacities and functional continuity [25,26]. This conceptual divergence poses a challenge for standardized monitoring and evaluation. Existing assessment tools for the IHR and health security—particularly the WHO Joint External Evaluation (JEE) and the World Organization for Animal Health (WOAH) Performance of Veterinary Services (PVS) Pathway—are central to the IHR Monitoring and Evaluation (M&E) Framework and are widely used to assess national preparedness and response capacities [27,28]. Although they assess may elements of preparedness capacities comprehensively, they lack robust indicators of long-term resilience and sustainability across sectors despite aiming to adopt a One Health approach [29].

[29] Moreover, they do not consistently account for how One Health coordination, environmental degradation, or donor dependency influence the durability of health systems.

As the IHR and Pandemic Agreement evolve, sustainability must be re-examined to ensure these tools drive equitable and durable capacity strengthening. To ensure the JEE and PVS evolve in line with contemporary priorities, it is critical to understand the perspectives of experts who design, implement and use them in planning, financing and decision-making contexts. This study therefore explores how global One Health and GHS experts perceive sustainability within the JEE and PVS tools and how these tools could be strengthened to better reflect evolving needs in a post-pandemic world. Accordingly, this study addresses three core research questions: (1) How do global One Health and GHS experts conceptualise sustainability within the JEE and PVS assessment tools? (2) To what extent do these tools currently operationalise such conceptualisations in practice across sectors? (3) How might sustainability be strengthened in future iterations of JEE and PVS to improve long-term, equitable capacity retention across human, animal, and environmental health systems?

## Methodology

### Ethics statement

This research utilised a qualitative approach involving individual interviews conducted through virtual video communication tools. Participants were provided with comprehensive information describing the purpose of the study, the interview process, and their entitlements, including the option to discontinue participation at any stage without any repercussions. Both written and verbal consent were secured before any data were gathered. Measures were taken to protect participant privacy by anonymising all data, and no personal identifiers are included in the reporting of results. Approval for the study was granted by the Ethics Review Committee of the London School of Hygiene and Tropical Medicine before the research began on 05/05/2023 (LSHTM Ethics Reference: 28748).

### Theoretical orientation and justification

This study was designed to examine how global One Health and health security experts perceive the usefulness and acceptability of existing sustainability indicators as reflected in the JEE and PVS tools. Given the study’s focus on subjective interpretation, context-dependent meaning, and expert reasoning, we adopted a social constructionist theoretical lens coupled with an interpretivist epistemology.

Social constructionism posits that knowledge—especially in the policy realm—is not a fixed or objective entity, but one co-produced through social processes, institutional practices, power asymmetries, and professional narratives [30–32]. In this view, concepts such as “sustainability,” “health security,” and “One Health” are not objective realities that are universally understood the same way, but constructed in different ways in different contexts, and by different disciplines and governance arrangements. A social constructionist lens enables researchers to analyze not just what is said, but how meanings are framed and legitimated through institutional and sectoral discourses.

Alternative paradigms were considered. Critical realism offers value when investigating generative mechanisms or causality within systems, while positivism assumes measurable, objective realities. However, both orientations aim to discover generalizable objective truths, while the main focus of our study was exploring diverse, subjective constructions of meaning [33]. For example, when trying to understand the impact of a health intervention like a new vaccination program, a critical realist approach might analyze its effectiveness in the context of wider determinants of health, such as access to healthcare, education levels, or income levels, and uncover how structural inequalities can undermine the intervention’s intended outcomes. A social constructionist approach might focus on how people perceive and understand the vaccine, its benefits, and the social meaning they attach to being vaccinated. They might explore how cultural beliefs, personal experiences, and social norms shape people’s decisions about vaccination. They would examine how the intervention is understood and interpreted within different social groups, potentially revealing how societal factors, like distrust in government institutions, can influence vaccination decisions. On balance, social constructionism aligned more directly with the study’s aim to examine how actors from different sectors, regions, and institutions *construct*, *contest*, and *negotiate* the idea of sustainability within global health security governance and the use of tools like the JEE and PVS [34,35].

### Sampling strategy and study population

The lead investigator (OAD), as a current member of both the One Health High Level Expert Panel (OHHLEP) and the World Bank Pandemic Fund Technical Advisory Panel (TAP), held an “insider” role. This afforded privileged access to ‘elite’ global expert networks and fostered trust and openness during the interviews. However, the lead researcher’s insider position within global One Health advisory spaces may have influenced interviewer-participant dynamics and recruitment. To address this, reflexive field notes were maintained, anonymity was guaranteed during data collection, and participants were explicitly encouraged to critique JEE/PVS systems to reduce social desirability bias. The implications of this positionality are addressed in the reflexivity statement (Supplementary information 1: Appendix 1), where strategies for mitigating bias are also described. A purposive sampling strategy was employed to ensure disciplinary, geographic, and institutional diversity among study participants covering the wide spectrum of human, animal and environment health. The sample was drawn primarily from three global technical expert platforms:

- OHHLEP, a multi-disciplinary group established by the Quadripartite (WHO, WOAH, FAO, UNEP) to provide independent technical and policy guidance on One Health [36];
- The World Bank Pandemic Fund Technical Advisory Panel (TAP), composed of global experts who review and score funding proposals based on their technical quality, sustainability plans, and adherence to One Health principles [37];
- Global One Health technical focal points from the Quadripartite institutions, responsible for operationalising the Joint Plan of Action (2022–2026) on One Health at national and international levels [38].

A small number of additional subject matter experts outside of these three groups were identified and recruited via snowball sampling (N = 5). Three invited experts were unavailable due to scheduling conflicts. No one declined to participate once invited. All interviewees were selected based on their cross-sectoral experience, with backgrounds spanning at least two of the human, animal, or environmental health domains. For descriptive purposes of the study, however, we classified interviewees based on their self-identified primary sectoral background. The final sample included 29 participants, with balanced representation across disciplines, sectors and income settings; 11 individuals with predominantly human health expertise, 11 with backgrounds in animal health, and 7 specialising in environment/ecosystem health. Alongside clinical and scientific disciplines such as human medicine, veterinary medicine, and the biological sciences, participants collectively brought knowledge from fields including epidemiology, statistics, health emergency response, logistics, social sciences, environmental engineering and management, political science, conservation, health economics, and mathematical modelling. The sample represented all major world regions—Africa, Asia, Europe, Oceania, and North and South America—and included professionals from both high-income settings and low- and middle-income countries (see Table 1). Gender was balanced across the sample frame with 15 female and 14 male participants. We do not report specific numbers by each category to assure the anonymity of interviewees. Environment specialists with combined global health security as well as One Health expertise were underrepresented in the sample despite concerted effort to recruit suitable participants, and reflects the wider underrepresentation of environment and ecosystem health specialists – particularly those hailing from LMIC settings – in global health security and One Health international policy fora.

**Table 1:** Participants’ characteristics according to primary sectoral background and national identity (World Bank country classification by income level).

Sector	High-Income Country	Low-/Middle-Income Country	Total (N)
Human Health	5	6	11
Animal Health	5	6	11
Environment/Ecosystem Health	6	1	7
Total	16	13	29

### Data collection and analysis

Data collection was conducted between July 2023 and March 2024. Informed consent was obtained verbally and in writing. All participants received, at least three weeks in advance, a 5-minute summary briefing derived from a prior document analysis of the JEE and PVS tools, conducted by the study team and published separately [29]. This document analysis applied an adapted version of the Schell et al. sustainability framework, identifying where and how sustainability was referenced across both tools [39]. A summary was also presented at the start of each interview via a PowerPoint slide set (Supplementary information 1; Appendix 2) to provide a shared entry point for reflection and discussion.

Interviews were conducted in English by two researchers experienced in qualitative research (OAD, MC) jointly, via Zoom or MS Teams video. Calls lasted 40–50 minutes and followed a semi-structured interview guide (Supplementary information 1: Appendix 3). All interviews were audio-recorded, transcribed verbatim and anonymized. MS Excel was used as a matrix-management tool to organise transcripts, codes, and mapped themes, while analysis followed Braun and Clarke’s reflexive thematic approach, enabling iterative comparison and theme development across sectors [40]. Three researchers (OAD, MC, HZ) independently reviewed transcripts and performed initial inductive open coding, identifying recurring patterns and emergent categories. This enabled multiple interpretations to surface and be captured – coding differences were discussed and resolved through consensus-building analytic meetings, with codebooks refined iteratively. In the second phase of analysis, we applied a structured coding frame that integrated constructs from both the Schell et al. sustainability framework and the Social Construction Framework (SCF) which support analysis through a social constructionist lens [34,35,39]. This dual-framework approach enabled us to link more tangible sustainability domains (e.g., financing, workforce, institutional infrastructure etc.) with discursive and power-related dimensions such as deservingness, framing, and narratives of accountability.

The analysis was iterative and interpretive. Line-by-line coding was conducted to ensure rigor and consistency. While doing this, one researcher (OAD) applied the constant comparative method, analyzing similarities and divergences across disciplines and income settings. Axial coding was used to group codes into higher-order thematic categories, and later interviews were used to test, refine, and validate earlier themes. Theme saturation was reached when no new constructs emerged, and at that point no more interviews were conducted.

The COREQ 32-item checklist was used to guide the reporting of this study’s qualitative methods and findings (Supplementary information 1: Appendix 4 in S1 File) [41].

## Results

Our analysis resulted in 7 key themes being identified when exploring participants views on measures of sustainability within current health security capacity assessment tools, namely those of the JEE and PVS. They included how sustainability is framed, its temporality, resources and ownership, building enduring capacity, One Health collaboration and power dynamics, equity and community engagement, and sustainability in policy and measurement. These themes are presented below with participant quotations supporting analytic interpretations.

### Framing sustainability: Process-oriented measures versus holistic outcomes

Across the interviews, experts commonly argued that the current GHS assessment tools frame ‘sustainability’ too narrowly, emphasizing the maintenance of systems and capacities at the expense of longer-term outcomes. Participants from all sectors noted that both the JEE and the PVS tool largely capture operational processes – such as laboratories functioning or protocols in place – but neglect the broader health and ecological results these systems are meant to sustain. This reflects a key concept in the Social Construction Framework (SCF), namely that certain capacities and sectors become “more visible” and thus more valued and resourced — while others (such as environmental health) are socially constructed as less deserving or lower political priority.) One senior veterinary expert observed *“the JEE and the PVS tend to focus on process-related aspects… rather than on broader sustainability outcomes”*, a sentiment echoed by counterparts in human and environmental health.

A physician interviewee noted that this reflects the origins of international health regulations prioritizing border security and trade over public health prevention, leading the tools to evaluate *“sustainability in terms of system processes… rather than measuring long-term health outcomes or ecological resilience.”* From an environmental health perspective, experts likewise criticized that current assessments rarely address long-term impacts on ecosystems or community well-being, instead checking whether capacities “remain operational” in the short term. Furthermore, the tools provide no clear definition of sustainability, and even the JEE’s sole mention of a timeframe – “a few years” – was deemed *“too vague”* by an animal health expert. Several interviewees argued that this narrow framing can create a false narrative of preparedness. As one public health expert cautioned, a country might meet the criteria for a “sustainable” capacity on paper while knowing that *“if donor funding decreases tomorrow, the system will collapse”* (human health expert). Such comments reveal a powerful narrative of accountability missing from the tools: they *“miss the deeper, long-term capacity that is needed, especially in lower-income countries where economic constraints are severe”* (human health expert). This demonstrates the SCF construct whereby LMICs are positioned as dependent recipients of external financial support, reinforcing a cycle where their sustainability is judged through externally imposed, narrow criteria and where self-sufficiency in financial terms is not valued in terms of sustainability. In short, experts across high- and low-income settings converged in critiquing the technocratic scope of existing metrics. Sustainability, they argued, should be re-framed beyond a checklist of capacities to encompass the enduring ability of those capacities to deliver public, animal, and environmental health outcomes under real-world conditions.

Notably, perspectives diverged on what additional dimensions should be included, reflecting each sector’s priorities. Human health experts (especially those from LMICs) emphasized the social and cultural context absent from the JEE/PVS. They noted that community trust, engagement, and local buy-in fundamentally affect whether capacities endure. For example, one interviewee highlighted that the tools: *“don’t address community engagement or the local priorities that affect how sustainable an intervention is in practice.”* (human health expert)

From the animal health side, economic considerations (such as the livelihood impacts on farmers or the veterinary workforce) were seen as underappreciated. Environmental health experts, in turn, were concerned that ecological sustainability – preserving ecosystem services and biodiversity that underpin health security – is virtually invisible in these assessments. One environmental specialist argued that sustainability of outcomes, like *“long-term human well-being, ecological resilience, or the economic and social impacts of interventions,”* is rarely examined in contrast to the narrow focus on sustaining processes. These differences in emphasis illustrate how power asymmetries in framing sustainability can marginalize certain sectors’ values: historically, human health and security concerns have dominated, casting animal and especially environmental health as secondary. The interviews reveal a growing consensus that truly sustainable health security must be constructed on a broader foundation. This involves expanding what counts as “success” beyond maintaining infrastructure – to include sustained disease control, improved population health, and even environmental regeneration and animal welfare as equally important outcomes of a resilient system.

### Temporal perspectives: Short cycles and the quest for long-term resilience

A second prominent theme was the question of timeframes – how long it takes to know if capacities are truly sustainable. Experts widely agreed that sustainability is an ongoing process rather than a one-off achievement, but they offered varying perspectives on the ideal assessment horizon, often reflecting their sector and context. Many participants felt that the JEE’s implicit timeline of just “a few years” is unrealistic for building durable systems. Veterinary and One Health experts from high-income countries (HICs) tended to advocate longer assessment windows to capture enduring change. For instance, one HIC animal health leader argued for a decadal perspective, noting that *“a minimum timeframe of 10 years is necessary”* to encompass *“multiple project cycles… and evaluate whether sustainable structures have been established and maintained”*.

By contrast, several experts with implementation experience in LMICs suggested a five-year cycle as a practical benchmark, aligned with common strategic planning periods and donor project durations. As one GHS advisor explained, a five-year period *“allows enough time to plan, implement, evaluate, and ultimately hand over projects”* – for example, transferring a new vaccine manufacturing capacity to full national ownership – and even then *“the possibility of extending to seven years”* might be needed for full sustainability (human health expert). This perspective underscores the importance of handover and transition in sustainability. Linking assessment timing to the rhythms of investment and political cycles was also a recurring idea. A public health official noted that while a five-year JEE cycle is acceptable in principle, its value is limited without synchronizing to real financing and support intervals. He suggested that:

*“subsequent assessments should be linked to a tangible investment cycle… to see if the investments and support provided have led to real, meaningful progress rather than simply checking a box every few years.”* (human health expert)

Participants across all sectors emphasised the need for adaptive and continuous monitoring models to account for fluctuating resources and political change. They argued that waiting five or ten years to revisit sustainability leaves too much to chance in between. One humanitarian expert favored *“iterative… annual evaluations”* to ensure that systems remain robust and to *“maintain pressure on stakeholders to keep improving”* rather than complacently declaring victory (human health expert). Environmental health specialists likewise endorsed a mix of short- and long-term indicators, balancing what is feasible to track frequently with what requires longer observation. For example, air or water quality metrics might be monitored annually, whereas ecological outcomes like biodiversity recovery might be assessed every 5–10 years (environment health expert). Crucially, this perspective introduced an equity consideration: countries with greater resources can afford more frequent, detailed evaluations, whereas resource-limited settings might need longer intervals between major reviews. As one environment expert noted:

*“the optimal timeframe is context dependent: richer countries might afford more frequent assessments, while resource-limited settings might need longer intervals… The key is to strike a balance without overburdening the system”*. Equity in sustainability monitoring thus emerged as a concern – an acknowledgement that one size may not fit all and LMICs for example, may not have the fiscal space for frequent reviews, meaning rigid expectations could unintentionally penalise those most at risk.

### Sustaining resources and ownership: Financing, politics, and the donor dilemma

Sustainable financing and local ownership were universally identified as linchpins of capacity durability. A clear theme was that without reliable, long-term funding and political commitment, any gains in health security are fragile – a truth often obscured by the tools’ cursory treatment of financing. Several experts pointed out a historical power asymmetry in GHS efforts: lower-income countries’ programs have been heavily donor-funded, raising concerns about dependency and deservingness. As one LMIC interviewee bluntly stated:

*“sustainability… must become part of the government’s core activities rather than a project funded by donors that vanishes when the money runs out.”* (human health expert)

Many shared this frustration that externally supported initiatives often fail to transition into nationally sustained programs, resulting in a cycle of pilot successes followed by collapse when donor priorities or budgets shift. The deservingness narrative of the SCF plays a subtle role here: governments and international partners may consider certain issues or regions ‘deserving’ of investment only as long as they align with donor interests, geopolitical interests, or crisis visibility, leaving other crucial capacities underfunded. An example raised was the post-COVID contraction of international aid; participants noted that sudden reductions in funding (for example, for disease surveillance or veterinary services) can rapidly erode capacities, which calls into question how “sustainable” they ever truly were. The interviews underscored that national budgetary commitment is the ultimate test of sustainability – capacities need to be institutionalized such that domestic resources and political will can maintain them.

From the perspective of global health experts, a related theme was the importance of crafting compelling narratives to influence power brokers – both domestic politicians and international donors – to keep resources flowing until sustainability is achieved. *“Governments and donors need a compelling justification for why health security investments should continue,”* one senior advisor insisted. *“This involves presenting solid data and demonstrating clear outcomes.”* (human health expert). This is both a practical strategy and a reflection of the power asymmetry in global health funding: low-income countries often must prove their worthiness to donors by showing results, and in turn, national health agencies must convince finance ministries to allocate domestic funds to health security capacities that may not show immediate payoffs.

Despite these challenges, the data also highlighted some positive examples of gradual shifts toward local ownership. In countries like Bangladesh, multi-year projects to establish capacities (e.g., vaccine production) are being designed explicitly with a handover in mind after about five to seven years, suggesting a model for donor support that builds in exit strategies suggested one human health expert. However, experts noted that the JEE/PVS assessments currently do not capture this dynamic of financing or transition. There was a strong call for embedding financing as a core indicator of sustainability – not only tracking the presence of plans and protocols, but whether there are dedicated budget lines, domestic co-financing, and plans for financial handover. As one interviewee observed, the JEE initially *“overlooked financing until it was later added”* as a component, and even now *“there is no rigorous follow-up mechanism to assess whether capacities remain sustainable over time”* (human health expert). Several participants therefore argued that sustainability metrics must explicitly include long-term financing levels and political support, to ensure that capacity building does not occur in a fiscal vacuum. In sum, the interviews paint a picture of sustainability as fundamentally a question of power and resources. A recurring warning was that without confronting the donor dependency cycle, GHS efforts risk remaining what one expert called:

*“a platform that only serves as an advocacy tool for donor support rather than genuinely strengthening the health system.”* (human health expert).

### Building enduring capacity: Workforce, knowledge, and institutional memory

When discussing what needs to be sustained in health security, nearly every expert pointed to human capacity and institutions as central pillars. The most tangible determinants of sustainability named across interviews were a well-trained workforce, strong institutions, and mechanisms to retain knowledge and skills over time. Participants repeatedly raised concerns about high turnover, brain drain, and the loss of expertise once initial training programs end – problems that plague both human and animal health systems, especially in low-resource settings. One LMIC researcher gave a striking example from her field: despite decades of infectious disease projects, *“there is a clear gap in transferring technical knowledge from one generation to the next”* in specialized areas like zoonotic disease research, meaning hard-won skills are not sustained (human health expert).

This underscores the need for institutional memory and succession planning. A One Health academic likewise emphasized *“investing in workforce development and ensuring effective succession planning”* as critical elements of sustainability (animal health expert). Notably, this challenge was often more acute in LMIC contexts, where reliance on short-term contracts and external trainings is common. Several participants highlighted structural workforce precarity — especially for early-career and contract-based technical staff — as a barrier to knowledge retention and One Health capacity sustainability. From an SCF perspective, the power asymmetry in knowledge was evident: international partners often deliver trainings, but local systems struggle to absorb and retain that expertise long-term, highlighting a need to shift power by strengthening local training institutions and career incentives.

To address these issues, experts advocated for strategies to anchor skills and expertise within countries and communities. Several interviewees stressed the importance of creating an environment where trained personnel remain engaged locally. *“It is essential to ensure that the people we train remain engaged with their local institutions so that the skills and knowledge do not dissipate after the project ends,”* explained one environmental health scientist, underlining that a sustainable system depends on retaining human capital. Incentives for retention might include clear career progression, adequate salaries, and recognition of expertise to dissuade talent from migrating to private sector jobs or abroad. This emphasises that sustainability is as much about human capital and job security as it is about technical capacity. From the environmental sector came suggestions to formalize this through capacity-building mandates – for instance, *“integrating mandatory One Health modules into training programs across medicine, veterinary, and environmental sciences”* to institutionalize cross-sector knowledge (environment health expert). Environmental and animal health experts in HICs further proposed that job descriptions and qualifications should explicitly include One Health and sustainability competencies, both to signal their importance and to ensure that incoming staff carry the needed ethos. One participant noted that if ministries required such competencies, it would *“serve as a powerful indicator of capacity”* and promote a *“culture of sustainability”* within the workforce (environment health expert). This idea of a “culture of sustainability” came up in multiple interviews – the notion that beyond technical skills, individuals throughout the system must internalize values of long-term thinking, collaboration, and stewardship. Cultivating this culture may involve continuous professional development and education. For example, human health experts suggested incorporating sustainability and equity principles into medical and public health curricula so that new professionals enter the field already attuned to these goals.

Another key aspect of enduring capacity is institutional infrastructure and governance to support the workforce. Strong institutions provide the stability and mandate for capacities to persist despite personnel changes or funding fluctuations. Participants highlighted the importance of clear institutional mandates (so that responsibilities for One Health or emergency preparedness are not ad hoc), robust legal frameworks that lock in commitments, and leadership development. One interviewee summarized the crucial elements as *“Stable funding and political support, robust legal and regulatory frameworks, strong organizational capacity and leadership, and integrated, multisectoral partnerships”* – arguing that without these, *“even the best-designed systems can collapse”* when circumstances change (animal health expert). In summary, the interviews underscored that sustainability rests on people and institutions as much as on plans and technology. Maintaining a skilled workforce, documenting knowledge, fostering a sustainability mindset, and fortifying institutions were all seen as interlinked prerequisites for any technical capacity (labs, surveillance, etc.) to endure.

### One health collaboration and power asymmetries: Balancing sectoral priorities

A strong consensus emerged that multisectoral collaboration – the essence of One Health – is itself a determinant of sustainability. Experts noted that siloed approaches in GHS are inherently fragile: if human health, animal health, or environmental health systems advance in isolation, they will eventually confront the limits of those silos when addressing complex threats. Therefore, sustaining capacity requires sustaining the relationships and coordination mechanisms among sectors. As one environmental health expert put it, *“health security strategies [must] embed a genuine commitment to intersectoral collaboration”*. Yet most interviewees also acknowledged significant power imbalances and value differences across sectors that complicate this ideal. A recurring observation was that human health institutions (ministries of health, in particular) tend to dominate decision-making and resources, often relegating veterinary and environmental agencies to a secondary status in practice. One senior veterinarian reflected that One Health collaboration *“must be built on mutual respect between sectors and a shared understanding of each other’s mandates”* – acknowledging that this mutual respect is not always present today. He gave a telling example: *“the animal sector’s focus on economic efficiency and disease control must be balanced with the human sector’s need for immediate healthcare and the environment sector’s emphasis on regenerative practices.”* Each sector brings its own priorities – economic livelihoods, direct health outcomes, or long-term ecological stewardship – and these can clash unless consciously reconciled. The social construction of each sector’s role influences whose priorities are seen as “deserving” attention. Historically, human health’s acute needs have been constructed as most deserving of urgent resources, while preventative veterinary measures or environmental protections were viewed as optional add-ons unless a zoonotic outbreak occurred. Experts from the animal and environmental fields often described narratives of marginalization, where their contributions are undervalued in national plans, policy fora, and donor agendas. Under SCF, environmental health actors in particular are socially constructed as lower power groups, leading to weaker influence and fewer sustainable investments in their domains.

Encouragingly, several HIC experts provided examples of shifting these dynamics through formal cooperation structures. In some cases, joint task forces and cross-agency agreements have started to equalize relationships. One veterinary institute head described how his institute established *“strong links with the human health sector through zoonotic disease programs”*, creating routine channels for data sharing and joint responses (animal health expert). However, he admitted *“we still lag in environmental integration,”* reflecting a common gap. Many noted that the environment sector remains the least institutionalized partner in One Health, often lacking an equivalent authority or clear mandate to participate, which is a power asymmetry rooted in governance. To address this, participants called for explicit integration of environmental expertise in health security decision-making – for example, including environmental scientists in outbreak investigation teams or as members of national IHR core groups. One interviewee suggested involving *“disease ecologists more deeply”* in preparedness planning, especially as climate change amplifies environmental drivers of outbreaks (environment health expert). Balancing sectoral interests, in their view, requires formal mechanisms that ensure each sector has a voice and resources commensurate with its role. This means health security plans must allocate some dedicated funding and staffing for animal and environmental health activities, not treat them as peripheral. As one expert argued, a truly holistic One Health strategy *“allocates resources equitably”* so that even if, say, the veterinary sector’s budget is smaller, *“its capacity-building and sustainable practices should not be neglected”* (animal health expert).

Another element repeatedly mentioned was the need for coordination platforms and cross-sector training to sustain collaboration. Joint simulations, inter-ministerial committees, and shared surveillance systems were cited as ways to keep sectors aligned. Some interviewees proposed that sustainability indicators should include measures of intersectoral collaboration – for instance, the frequency of joint outbreak drills or the existence of data-sharing agreements. From a social standpoint, building personal relationships and trust among sector leaders was seen as invaluable. In one striking suggestion, a GHS expert recommended appointing a high-level One Health champion or coordinator within government to act as a bridge and *“advocate across boundaries”* (human health expert). This role could help mediate conflicts – for example, between a health ministry focused on human disease control targets and an agriculture ministry focused on trade impacts of livestock diseases – and find win-win narratives. Indeed, several participants stressed crafting a narrative of co-benefits: communicating that improving veterinary services also reduces human outbreak risk and protects livelihoods, or that environmental measures (like water sanitation) yield health benefits. As one interviewee explained, *“Balancing sectoral interests requires clear communication of the co-benefits for all parties… so that the advantages for each sector are articulated and understood from the outset”* (environment health expert).

### Equity, community engagement, and decolonizing global health security

Interwoven through the above themes is a set of cross-cutting issues – equity, community engagement, and decoloniality – that experts argued must be addressed to achieve true sustainability. The majority of interviewees, especially those from LMIC backgrounds, pointed out that GHS initiatives often fail to incorporate local voices and context, reflecting a top-down legacy. Using an SCF lens, there was a palpable concern around issue framing and power and that without correcting these imbalances, sustainability efforts would perpetuate existing disparities between countries and between institutions and communities. One of the stark critiques was the absence of community-level engagement in current assessment tools. Participants observed that the JEE and PVS do not evaluate whether interventions are culturally appropriate, accepted by communities, or adapted to local needs – factors which determine if those interventions will stick. One LMIC expert lamented that *“the tools rarely capture the socio-cultural context. They don’t address community engagement or the local priorities”*, effectively treating communities as passive recipients rather than active architects of sustainability or health security (human health expert). Participants noted that excluding local knowledge and indigenous practices undermines long-term continuity — as sustainability depends on cultural acceptance and local agency. An environment health researcher recounted a telling experience: in one project, his team invited local pastors and community leaders to discuss their surveillance findings, which greatly improved community understanding and buy-in, yet *“this kind of community engagement… is not consistently captured by the assessment tools.”*

Several experts argued that this reflects a lingering colonial mindset in GHS – where external ‘experts’ design solutions with little input from those on the ground, and success is measured by external standards rather than community-defined benefits. To counter this, interviewees advocated a more equitable and inclusive approach at both national and international levels. At the national level, this means engaging community stakeholders, local governments, indigenous groups and frontline workers in planning and evaluation. A recurring suggestion was to integrate community feedback and indicators of community trust or involvement into sustainability assessments for health security. Environment health experts emphasized that affected communities often possess critical knowledge about local environmental risks and resource management; thus, empowering these voices can improve the design and longevity of interventions. One interviewee stressed that underrepresented groups must be brought into decision-making for health security, saying sustainability is achieved only when *“the voices of all stakeholders—including underrepresented communities…—are heard and integrated into decision-making”* (environment health expert). This principle aligns with the broader movement to decolonize global health, which calls for shifting power and agency to local actors. In the context of One Health, decoloniality would involve valuing indigenous and local know-how (for example, farmers’ knowledge of animal diseases or traditional ecological practices) on par with external expertise, and ensuring local stakeholders have ownership of health security programs.

At the international level, equity concerns were raised regarding how the JEE and PVS are implemented and used. Some LMIC experts subtly noted the power imbalance in the evaluation process itself: assessments often involve international teams and benchmarks that may not fully consider local constraints or engage community representatives in the assessment process. There is a sense that low-income countries are under the microscope to prove their capacities according to external criteria, which can reinforce a narrative of deficit and dependency. True sustainability, from a decolonial perspective, would mean that countries define their own indicators of progress (tailored to their context) and engage in mutual accountability with external partners, rather than one-sided scrutiny. The recent emphasis on equity in pandemic preparedness negotiations was mentioned as a hopeful sign – a chance to embed fairness (e.g., equitable access to resources and voice) as a core part of GHS. Interviewees underscored that donor influence must be wielded responsibly to support country-led priorities instead of imposing agendas. Several participants called for donors and international agencies to re-frame their expectations of success: rather than only counting labs built or trainings held, they should value measures like community satisfaction, reduction in disparities, and empowerment of local institutions. This reframing ties back to the SCF idea of deservingness – expanding it to assert that all communities and sectors (not just those in high-profile outbreaks or urban centers) deserve sustained investment and capacity.

### Embedding sustainability in policy and measurement

Finally, participants discussed how sustainability could be made an integral, measurable part of health security planning and evaluation going forward. The consensus was that both national strategies and international assessment tools need to be explicitly reoriented to include clearly defined sustainability criteria, so that the concept moves from rhetoric to operational reality. Several experts noted that recent policy developments (like the 2024 IHR amendments and the Pandemic Agreement drafts) call for sustainability and equity, but these high-level commitments must translate into concrete indicators and accountability mechanisms. Addressing narratives of accountability from an SCF perspective, many interviewees therefore offered recommendations on what to measure and monitor in order to capture the process and outcomes of sustainability. A senior global health security evaluator observed that progress is being made – for example, *“the initial iterations of the JEE… overlooked financing until it was later added”*, a gap that is now recognized. However, he emphasized that *“even now, there is no rigorous follow-up review mechanism to assess whether capacities remain sustainable over time”* (human health expert). Regular tracking of *“ongoing financing levels”* was thus proposed, to see if domestic funding is increasing to gradually replace donor funds (human health expert). These kinds of indicators were seen as vital to detect whether a country’s capacity is improving, plateauing, or deteriorating post-assessment.

Experts from all sectors strongly supported the idea of dual indicators for process and outcome – mirroring the earlier theme that both the existence of systems and their real-world impacts matter. On the process side, interviewees recommended indicators of institutional capacity and governance, such as *“whether national plans include sustainability training for staff, or whether multi-sector coordination committees meet regularly.”* (animal health expert). The presence of policies like maintenance plans for equipment, workforce succession plans, or community engagement strategies could also serve as process indicators of sustainability-minded governance. On the outcome side, suggested metrics included long-term trends in disease incidence (to see if capacities lead to sustained risk reduction), the resilience of wildlife or livestock populations, and even broad measures like ecosystem health in areas targeted by One Health interventions. One interviewee suggested incorporating *“indicators related to climate change, ecosystem health, and even aspects of wastewater management”* into national health security monitoring, to ensure environmental drivers are not forgotten (environment health expert). Importantly, multiple participants highlighted accountability and enforcement metrics as part of sustainability. It is not enough to have plans on paper; there should be indicators like *“the frequency of compliance audits or regulatory actions”* taken to uphold standards. (environment health expert).

Another recurring recommendation was to build transparent data systems and feedback loops for sustainability. Several experts across sectors argued for open-access data dashboards where key sustainability indicators for health security are published regularly. This would allow not only governments but also researchers, civil society, and international partners to see how capacities evolve. *“Equally important is the need for open data systems that allow all stakeholders… to access and analyze performance data,”* one interviewee noted, *“Only by creating transparent feedback loops can we ensure that sustainability remains a central, evolving component of health security planning.”* (environment health expert). Transparency was seen as a means to drive mutual accountability. Some even suggested that international financing could be tied to these indicators, rewarding countries that maintain capacities and providing support when slippage is detected – an idea aligning with the concept of a Pandemic Fund that incentivizes preparedness. Consistent definitions and benchmarks were also deemed crucial. For instance, what qualifies as “sustainable workforce capacity” needs to be defined so that assessments are comparable over time and across teams. *“Consistent definitions and benchmarks would help ensure that if one team assesses sustainability today and another does so a year later, the results will be comparable,”* explained one expert, underscoring that without standardization, sustainability scores or ratings can be overly subjective (human health expert). This speaks to the need for the WHO, WOAH, and partners to refine the JEE/PVS guidance to clearly define sustainability elements (like financial sustainability, community ownership, etc.) and how to rate them.

Finally, participants emphasized embedding these indicators into national planning cycles and policy instruments, not just external evaluations like the JEE or PVS. Many advocated that countries include a dedicated sustainability section in their national action plans for health security or One Health strategies. This section would outline how each capacity built (e.g., a laboratory network, a surveillance program) will be maintained long-term – covering financing, training, community engagement, and so on – along with indicators to monitor those aspects. By doing so, sustainability becomes a *“foundational principle rather than an afterthought”* in planning (animal health expert). Some even suggested writing sustainability requirements into legislation or regulations, to ensure continuity beyond individual projects or political administrations.

As shown in Table 2, process versus outcome orientation, financing, and timeframes were framed by interview respondents differently across sectors, and between HIC and LMIC settings. Differences in perceived “deservingness,” power, and visibility or accountability across sectors align with the SCF, influencing which capacities are prioritised for sustained investment.

**Table 2:** Summary of Sectoral and Contextual Contrasts in Sustainability Framing.

Dimension	Human Health	Animal Health	Environmental Health	LMIC-Specific Considerations	HIC-Specific Considerations
Framing of Sustainability	Systems continuity and service delivery	Livelihoods, biosecurity, economic resilience	Ecosystem health, biodiversity, intergenerational equity	Dependency on donor financing; fragility of systems	Long-term planning; institutional memory
Key Challenges Identified	Workforce retention; community trust	Economic externalities; underfunding	Marginalization in health security discourse	Political instability; weak local ownership	Fragmented responsibilities across sectors
Timeframe Considered Adequate	5–7 years aligned with strategic cycles	10 + years to capture structural change	Mixed: annual monitoring + long-term trend evaluation	Funding cycle dependent; capacity building needed	Regular reassessments feasible; better resourced
Sustainability Emphasis in Tools	Under-developed; weak community indicators	Minimal ecological/livelihood measures	Virtually absent from current tools	Tools do not reflect LMIC-specific sustainability risks	Existing capacities better aligned with assessment

## Discussion

This qualitative study reveals that the current framing of sustainability within GHS assessment tools – namely the JEE and PVS – remains narrowly process-oriented and insufficiently aligned with long-term outcomes, contextual realities, or One Health imperatives. Across all sectors, experts critiqued the absence of robust mechanisms for capturing the durability of capacities, particularly in LMICs where donor dependency, workforce attrition, and institutional fragility undermine sustainability. These insights resonate with critiques from the existing literature, which note that although revisions of the JEE and PVS have incrementally improved in scope, they still fall short in embedding sustainability as a structural goal rather than a procedural achievement [24,25,42]. Notably, several participants argued that sustainability cannot be divorced from the global political economy of health security governance. The severe funding contractions from major donors — including the UK FCDO and USAID — since 2020, and the increasing securitisation of global health under “national interest” doctrines, were viewed as existential threats to the durability of capacities in LMICs. This reflects a recurrent criticism of IHR implementation: without guaranteed, long-term, and sovereign financing, sustainability remains aspirational [12–14,42].

Sustainability was widely understood by participants not merely as the continued existence of systems, but as the enduring capacity of those systems to adapt, function, and deliver meaningful outcomes across human, animal, and environmental health. In contrast, earlier iterations of the JEE and the PVS Pathway were designed primarily as snapshots of technical capacity and legal compliance, reflecting a technocratic model of preparedness [43,44]. Although the 2024 amendments to the IHR acknowledge One Health and sustainability explicitly for the first time, operationalising these commitments through measurable, sector-specific indicators remains a gap [6,45].

### Sectoral and contextual differences

A key contribution of this study is its detailed elucidation of sectoral and contextual divergences in how sustainability is understood. Human health respondents from LMICs foregrounded issues such as workforce retention and local ownership, while environmental health participants underscored ecological resilience and intergenerational equity. Experts in the animal health sector stressed economic externalities, such as the fiscal vulnerability of veterinary services in agricultural economies. These differences are consistent with the findings of sustainability literature captured in recent systematic reviews, which report that determinants of sustainability are often domain-specific but converge on broader systemic enablers such as leadership, institutionalization, financing, and stakeholder alignment [24,46,47]. These divergences also align with the SCF, wherein certain sectors (particularly human health) are socially constructed as more “deserving” of sustained investment than others. The relative marginalisation of environmental health capacities — despite their foundational role in zoonotic prevention — exemplifies how power dynamics shape which determinants are prioritised.

Findings from this study reaffirm that sustainability cannot be addressed in a monolithic fashion. Rather, the meaning and determinants of sustainability differ across sectors and are profoundly shaped by contextual realities such as funding landscapes, political systems, and social norms. For example, human resource sustainability emerged as a cross-cutting vulnerability. Interviewees emphasised the emotional toll, occupational risk, and post-emergency job insecurity experienced by frontline personnel, with inadequate compensation systems leading to attrition and brain drain. Without reforms that secure terms of employment, protect health workers, and recognise epidemiology, veterinary, and environmental science career pathways, sustainability becomes fragile, particularly in LMICs where institutional memory is easily lost.

### Evolving global frameworks and institutional mandates

The 2024 amendments to the IHRs, alongside the drafting of the Pandemic Agreement, represent a watershed moment for reform and redefining sustainability in GHS. The inclusion of equity, access, and One Health principles reflects a broader shift toward structural resilience, rather than merely response readiness [45,48]. However, integration into national systems and evaluation tools remains underdeveloped.

Parallel to this, the One Health Joint Plan of Action (OH JPA) and its Implementation Framework—spearheaded by the Quadripartite collaboration (WHO, FAO, WOAH, UNEP)—offer a blueprint for operationalising sustainability via intersectoral coordination, community engagement, and environmental stewardship [38]. Interviewees noted that OH JPA’s emphasis on capacity building, governance, and integrated service delivery aligns well with the proposed sustainability constructs emerging from this study, yet there is currently no formal mechanism linking JEE or PVS indicators with OH JPA implementation benchmarks.

The ongoing negotiations around the Pandemic Agreement provide both opportunity and caution. On one hand, the agreement could institutionalize global commitments to sustainable preparedness financing, thereby enabling LMICs to transition from donor dependency to fiscal autonomy. On the other, failure to establish equitable financing mechanisms may entrench existing disparities [7,48]. The widespread funding cuts to global health and development over the last four years since the COVID-19 Pandemic—documented across the UK FCDO, USAID, and other bilateral donors—have already led to the closure of programs, disruption in medicine supply chains, and collapse of key capacities in LMICs [49–51]. These conditions reinforce the urgency of integrating sustainability indicators that account for financing pathways, national co-investment, and budgetary resilience within the assessment frameworks of global health security. Several LMIC participants further highlighted the need to re-balance global governance structures so that sustainability priorities are not externally imposed but generated through domestic political processes. This includes strengthening fiscal sovereignty, integrating sustainability into national legislation, and resisting conditionalities that undermine long-term planning. The recent launch of the America First Global Health Strategy underscores the importance of the issue with a major global donor now overtly predicating significant financing of global health on the use of its own products – no different in essence from a government subsidy for industry – through transactional bilateral arrangements that potentially undermine both data and specimen sovereignty as well as the development of homegrown manufacturing in LMICs of medical countermeasure products like diagnostics, vaccines and therapeutics [52].

### Advancing sustainability metrics: A proposed framework

Drawing on both interview data and systematic review literature, we propose a high-level schema of sustainability determinants that can be more explicitly embedded into future iterations of JEE, PVS, and related IHR M&E tools and framed as such. These are outlined in Table 3 below. Importantly, several of these indicator categories—particularly those related to financing and investment (e.g., existence of budget lines for health security), workforce and capacity retention (e.g., tenure and turnover rates), institutionalization and governance (e.g., presence of multisectoral coordination bodies and legal mandates), and community engagement (e.g., mechanisms for community input and feedback)—are already being collected in routine surveillance systems, national health plans, and WHO’s JEE and SPAR mechanism. PVS Pathway reports also routinely capture institutional and workforce capacities but do not necessarily explicitly refer to these indicators as relating to sustainability [3,27,28,44]. Systematic reviews confirm that these determinants are traceable and have been repeatedly highlighted as core pillars of sustainability [46,47]. The World Bank/WHO Global Preparedness Monitoring Board (GPMB) has similarly emphasized the need to leverage existing data sources and national planning instruments to reduce the burden of measurement while improving accountability [44].

**Table 3:** Proposed Core Determinants and Domains of Sustainability Measurement for JEE, PVS, IHR and One Health Assessments.

Determinant Domain	Category of indicators (examples)	Relevance across sectors	Income Setting considerations and contrasts
**Financing and Investment**	Long-term budget lines, domestic co-financing	All	LMICs: donor exit strategy; HICs: fiscal accountability
**Workforce and Capacity Retention**	Staff tenure, career progression, succession planning	Human, Animal	LMICs: brain drain; HICs: institutional memory
**Institutionalization and Governance**	Legal mandates, embedded plans, multi-sectoral leadership	All	LMICs: political fragility; HICs: decentralized systems
**Community Engagement and Trust**	Participatory planning, local feedback mechanisms	Human, Environmental	LMICs: social legitimacy; HICs: accountability
**Environmental and Ecological Health**	Biodiversity monitoring, ecosystem services integration	Environmental, Animal	LMICs: natural resource dependence; HICs: compliance focus
**Cross-sectoral Collaboration**	Frequency of inter-ministerial meetings, joint action plans	All	LMICs: power asymmetries; HICs: siloed mandates
**Temporal Durability**	Measurement across strategic cycles (5, 10 years)	All	LMICs: adaptive planning; HICs: programmatic alignment

### Implications for policy and practice

The findings from this study point to three key policy implications. First, GHS frameworks must shift from short-term process audits toward long-term sustainability planning, particularly in LMICs where external support often substitutes for systemic resilience. Second, multisectoral equity must be structurally embedded in evaluation criteria—ensuring that environmental and animal health perspectives are not marginalized in ostensibly One Health assessments. Third, international financing facilities, such as the Pandemic Fund, should explicitly link disbursements to progress in sustainability planning and locally owned capacity transitions. This is especially important given emerging shifts toward more transactional, donor-interest-driven global health — evidenced by recent strategies prioritising national gains over collective risk reduction which further challenges assumptions that external financing can be relied upon to sustain core capacities [52].

To support these implications, WHO and WOAH could revise the JEE and PVS tools to include a dedicated “Sustainability and Equity” technical area, tied more closely to benchmarks derived from the OH JPA and the GPMB’s calls for systemic preparedness investments [35,41]. Indicators could be co-designed with country partners, reflecting context-specific priorities, financing capacities, and data availability. For example, several interviewees in our study noted that indigenous and other local knowledge systems remain largely absent from current sustainability indicators. Ensuring that environmental and zoonotic surveillance systems capture — and protect — indigenous knowledge could support more community-owned, context-appropriate sustainability measures and enhance data systems for future digital and AI-enabled tools [53].

### Study limitations

This study is limited by its reliance on ‘elite’ expert informants, many of whom operate at international or institutional levels. While this affords insight into the design and interpretation of assessment tools, it may under-represent local operational and community perspectives. The positionality of the lead researcher as an insider to several global governance arenas may also have shaped access and the dynamics of interviews; this was mitigated through reflexive documentation (see reflexivity statement) and explicit assurance of anonymity to participants. In addition, interviews were conducted in English and via virtual platforms, which may have excluded experts less comfortable with these modalities. However, purposive sampling ensured a breadth of disciplinary, geographic, and institutional perspectives. In addition, and reflecting the wider under-representation of the environment sector in the GHS and One Health policy arenas, the research team found it relatively more difficult to recruit environment health experts with both international experience and expertise in One Health and GHS to the study – especially those from LMICs – this was mitigated by having an extended data collection period and through our combined purposive and snowballing sampling strategy.

### Recommendations for future research

Future work should include co-production of indicators with national governments and civil society, particularly from the environmental sector, which remains underrepresented. Operational research linking sustainability ratings to real-world health emergencies and outbreak outcomes could provide empirical validation for revised frameworks. Finally, economic modelling and return on investment analyses to estimate the cost–benefit of sustainability investments—particularly in relation to donor transition and local fiscal absorption—could help build the financial case for reform, strengthen the application of the One Health approach over the long-term, and ultimately foster local ownership of health capacity building initiatives.

In conclusion, we recommend that future iterations of health security capacity assessment tools like the JEE and PVS clearly define sustainability and its determinants for their purposes. They should also incorporate explicit sustainability metrics, aligned with the revised IHR, the One Health Joint Plan of Action and relevant SDG targets, as well as ensure these are sensitive to national planning cycles. Tools must also reflect sectoral and contextual nuances, and integrate long-term monitoring frameworks that promote domestic accountability and local ownership. In the field of One Health and GHS, strengthening the sustainability components of these tools is essential to build equitable and resilient health systems globally. Ultimately, sustainability in GHS is a question of sovereignty: capacities will endure only when countries retain political and economic control over the systems designed to protect their populations.

## Supporting information

S1 File Appendices. (DOCX)
