# Supplementary material for: Exploring measures of sustainability in the WHO Joint External Evaluation and the WOAH performance of veterinary services tools—A qualitative assessment of perceived usefulness and acceptability to one health and global health security experts
Source: PLoS One. 2026 Mar 4;21(3):e0343801. doi: 10.1371/journal.pone.0343801 (PMC12959694; doi:10.1371/journal.pone.0343801)
Supplement: S1 File — (DOCX) [file pone.0343801.s001.docx]

**Supplementary Information 1**

**Appendix 1: Reflexivity statement**

**Researcher Characteristics and Positionality**

The study was designed and led by a primary investigator (OAD) with a professional background in global health and emergency public health, including extensive experience working at the intersection of policy, research, and practice in low-resource and humanitarian settings. OAD is a current member of two high-level global expert platforms: the One Health High Level Expert Panel (OHHLEP) and the World Bank Pandemic Fund Technical Advisory Panel (TAP). This professional positioning afforded the researcher privileged access to global expert networks and contributed to the trust and credibility perceived by participants. However, this “insider” status also required careful reflection on potential sources of bias. OAD’s established relationships with many participants may have influenced how openly views were shared—both positively, by fostering candour and trust, and negatively, by introducing potential desirability bias or professional deference. To mitigate this, efforts were made to maintain an open and non-hierarchical interview atmosphere, encourage critical viewpoints, and emphasize the voluntary and confidential nature of participation. Interviews were conducted jointly by OAD and another researcher (MC) to encourage further openness and reduce the risk of bias.

Three researchers (OAD, MC and HZ) from the study team contributed most to the design and analysis. These researchers brought complementary disciplinary backgrounds in public health, one health and social science, with no direct prior affiliations with the expert platforms involved. Their external perspectives were essential for challenging assumptions, enhancing analytic rigor, and enabling iterative discussions around theme development.

We acknowledge that all members of the research team hold certain epistemological orientations aligned with interpretivism and policy-informed social science. While this provided cohesion in the methodological approach, it also necessitated vigilance against confirmation bias during data interpretation.

**Interviewee Characteristics and Power Dynamics**

The study population consisted of 29 participants drawn from leading global technical and policy bodies. Participants were selected based on their affiliation with the OHHLEP, Pandemic Fund TAP, and/or their roles as technical focal points in the Quadripartite organizations (WHO, WOAH, FAO, UNEP). These individuals are recognized as subject matter experts in human, animal, and environmental health, with significant influence in shaping global health security and One Health policy.

Interviewees included 11 experts from a human health background, 11 from animal health, and 7 from environmental or ecosystem health disciplines. Sixteen participants were from high-income countries and 13 from low- and middle-income countries. All had extensive cross-sectoral experience and occupied roles that span research, implementation, donor engagement, and policy development.

Given the seniority and influence of many participants, power asymmetries between researchers and interviewees were not unidirectional. In fact, the researcher–participant relationship often involved mutual recognition as peers or collaborators within overlapping global health networks. These dynamics had the potential to influence both the willingness to disclose critical views and the interpretation of language and emphasis during interviews.

To address this, interviews were structured around open-ended, reflexive questions that encouraged participants to articulate their own definitions and experiences without prescriptive framing. The use of a summary document (based on prior tool analysis) presented prior to the interview helped establish shared reference points but did not constrain the content of discussion. Interviewees were explicitly invited to critique, refute, or expand on the framework presented.

**Analytic Reflexivity and Interpretation**

Thematic analysis was undertaken with an awareness of how our disciplinary perspectives and professional experiences could shape theme identification. For instance, the integration of both the Schell et al. framework (focused on operational sustainability domains) and the Social Construction Framework (focused on discursive and political dimensions) was designed to capture a broader range of meanings—but also reflected the research team's interest in systems and power.

To enhance interpretive rigor, data coding and theme development were conducted collaboratively and iteratively across team members with varied disciplinary orientations. In addition to the lead investigator (OAD), who has a global health and emergency public health background, the team included MC, a One Health specialist from a High-Income Country (HIC), and HZ, a global health professional with field-based experience in humanitarian health response in low- and middle-income country (LMIC) settings. These disciplinary and experiential differences were valuable for ensuring analytic pluralism. MC contributed expertise in multisectoral program design and inter-institutional collaboration, while HZ brought a grounded understanding of how sustainability is operationalized in fragile settings. Their perspectives enriched the interpretive process and enabled deeper insight into how the framing and experience of sustainability might differ across technical domains and geographic contexts.

Disagreements in interpretation were treated as opportunities for deeper analytic reflection rather than resolved through majority consensus. Where possible, emergent findings were triangulated across different sectors, geographies, and professional domains, and themes were tested in later interviews for consistency and resonance.

While the insider-outsider composition of the team helped mitigate individual biases, we acknowledge that complete neutrality is neither possible nor desirable in qualitative inquiry. Instead, we approached this study with transparency, humility, and a commitment to interpret the data in a manner that was faithful to participants' voices and experiences while recognizing the broader institutional and political contexts in which they operate.

**Appendix 2: Pre-interview document analysis slide deck**


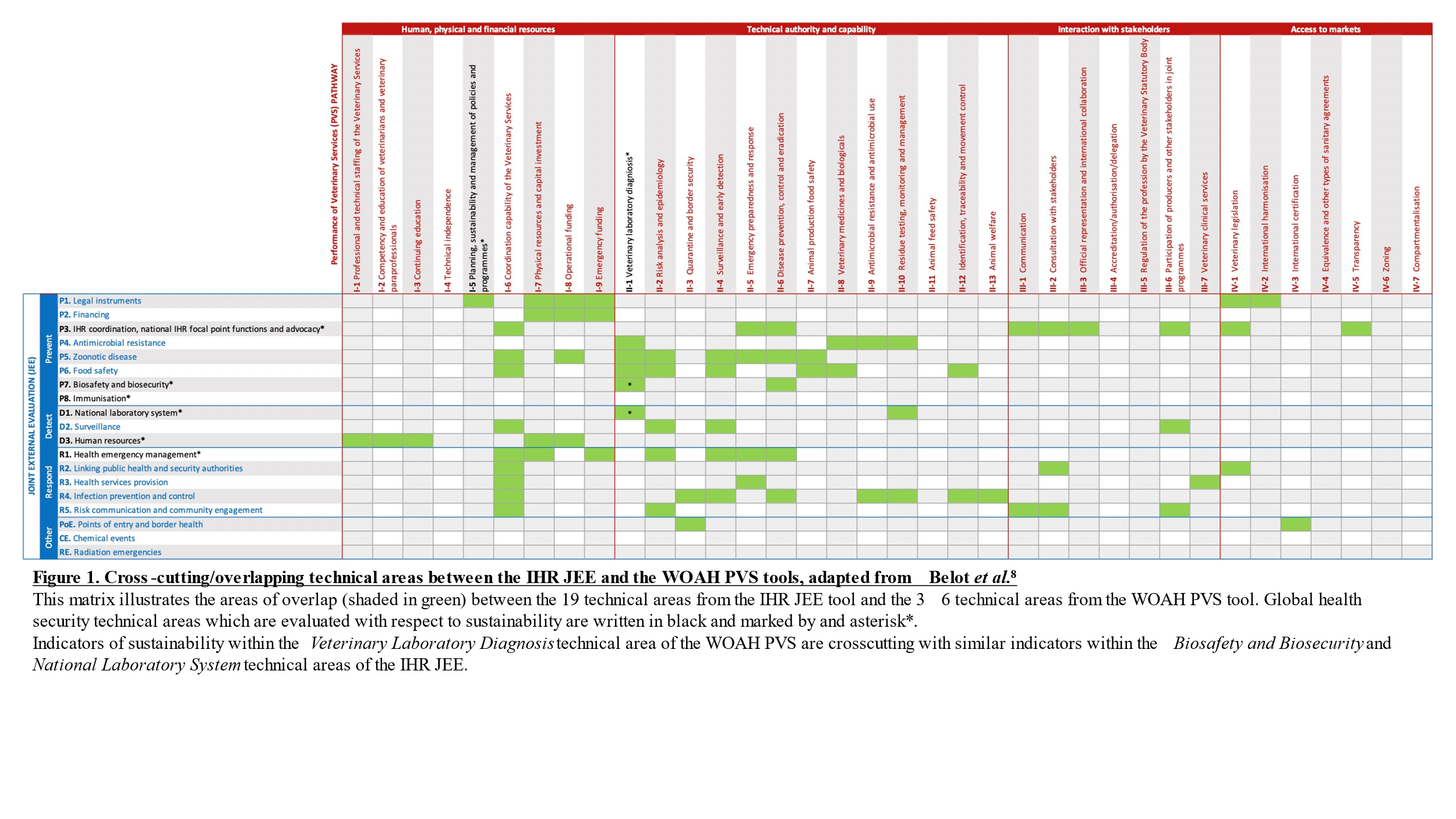


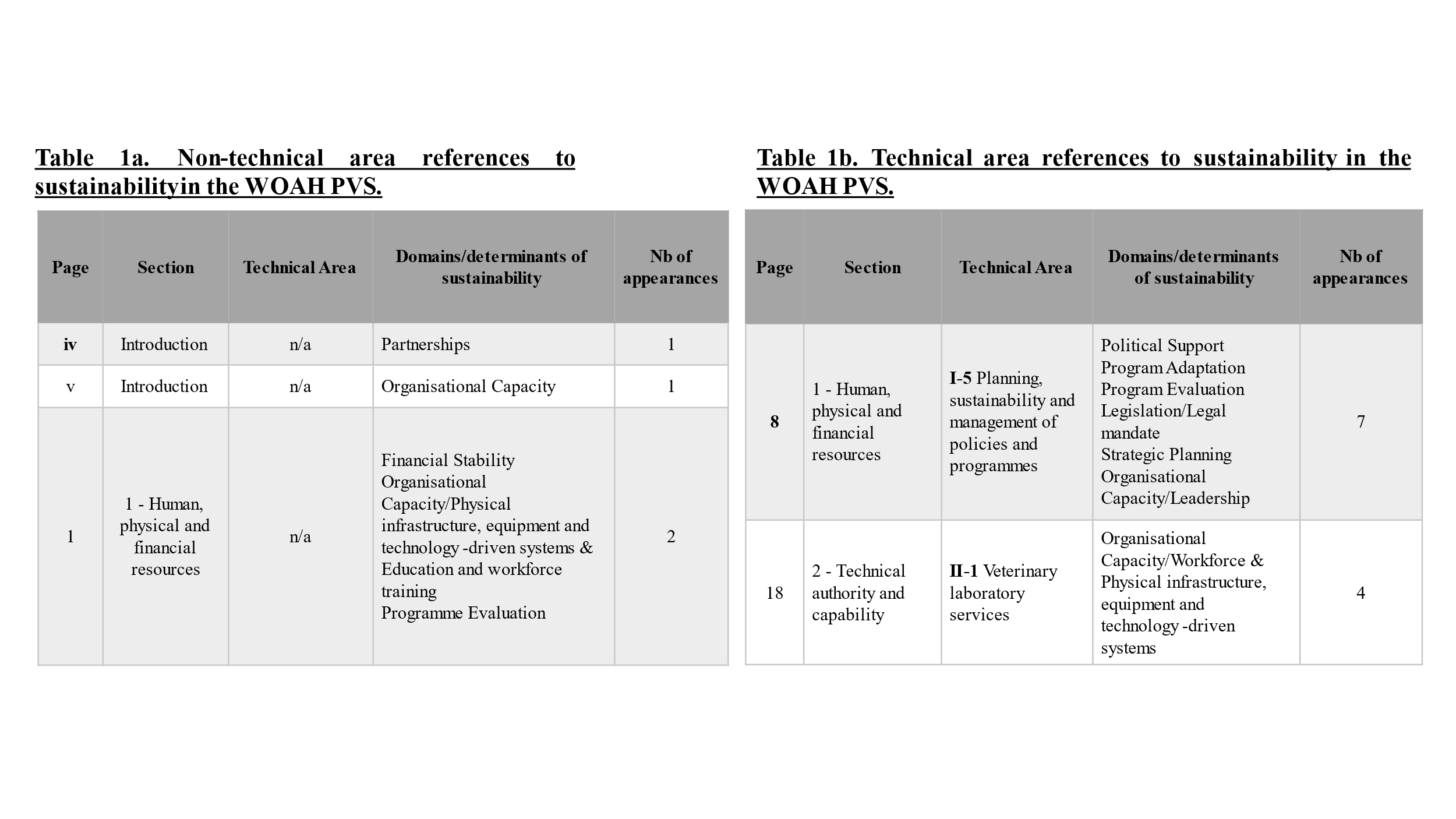


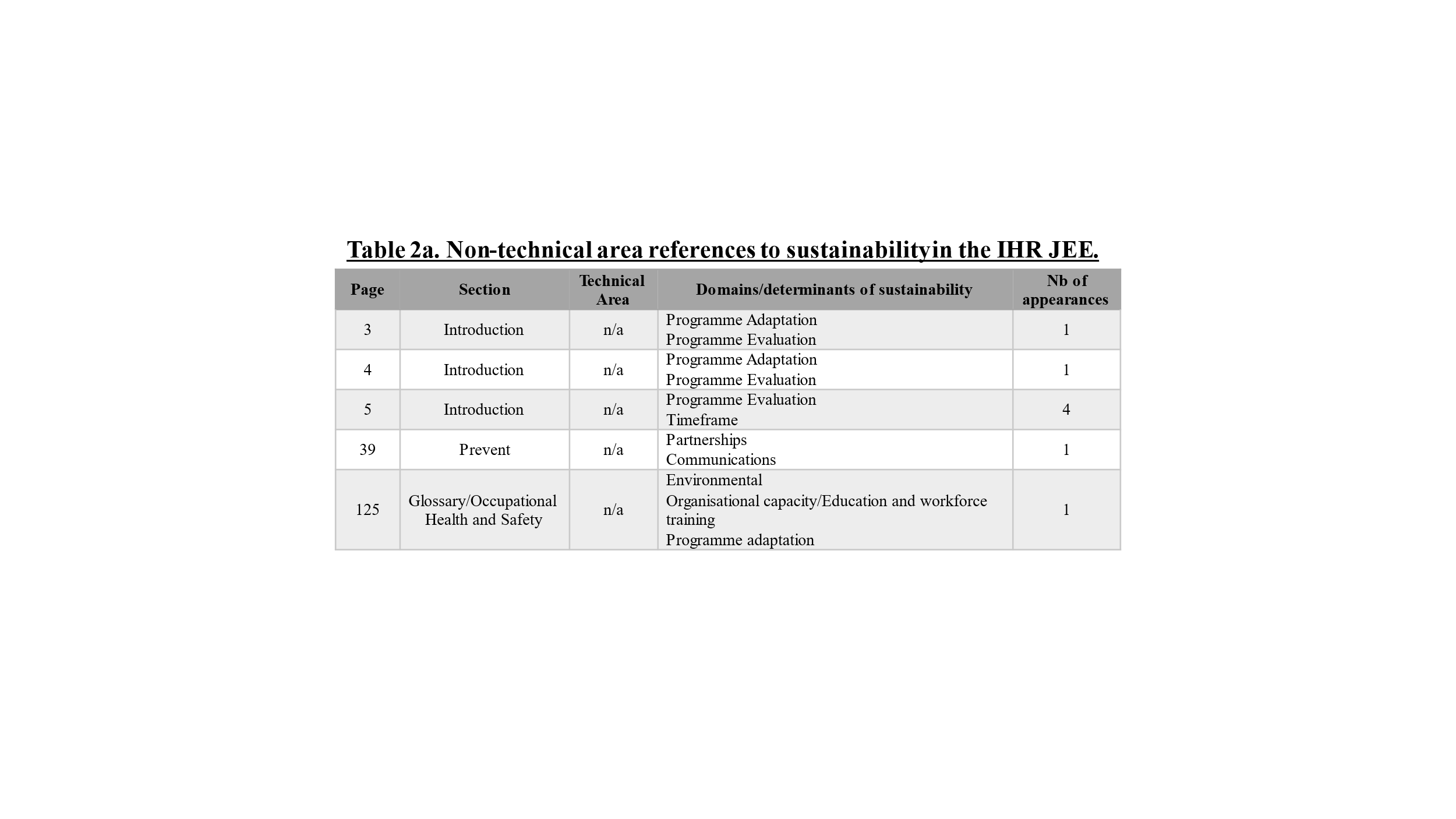


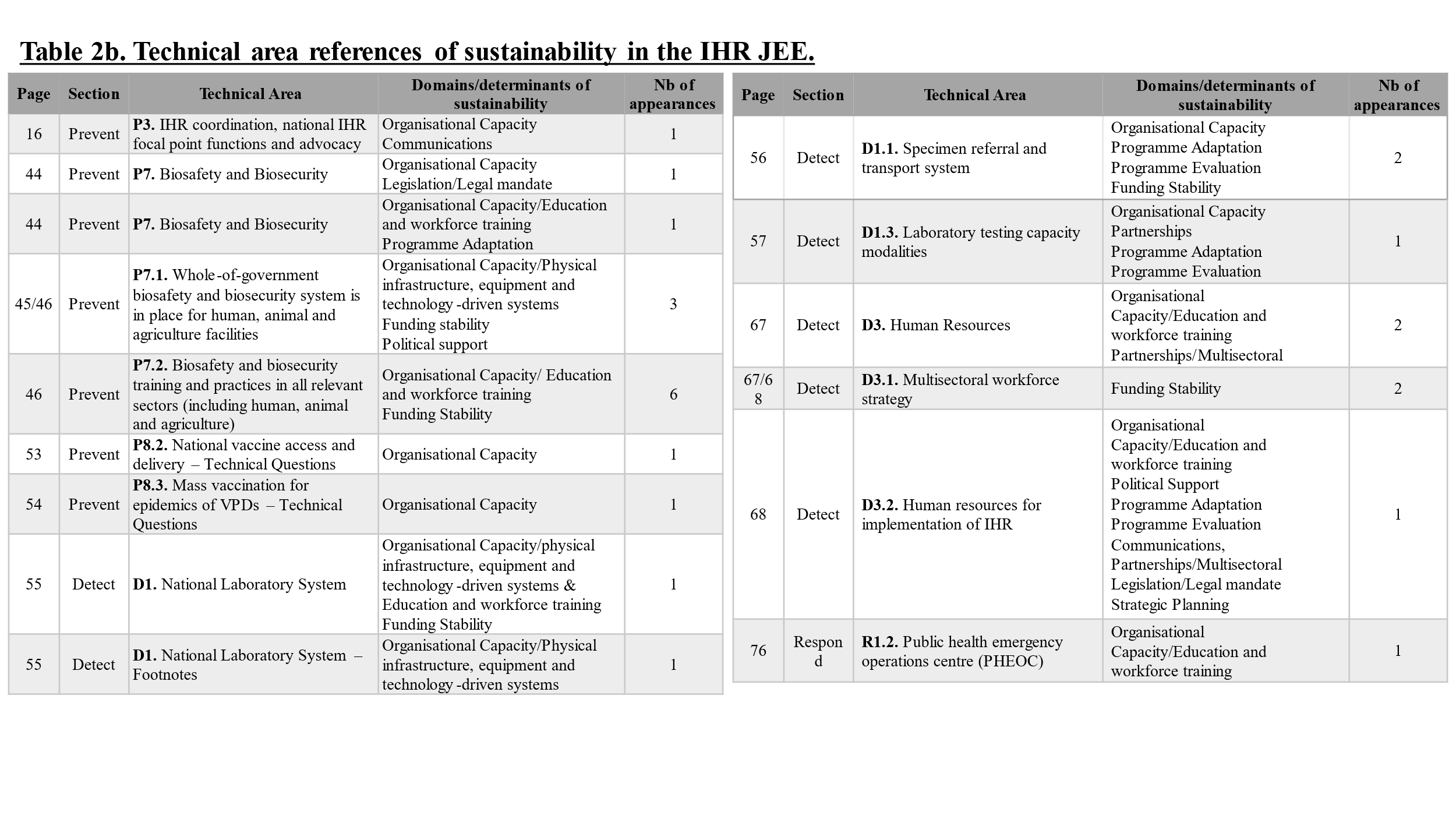


**Appendix 3: Semi-structured interview topic guide**

**Study title***: Exploring measures of sustainability in the WHO JEE and the WOAH PVS tools – a qualitative assessment of perceived usefulness and acceptability to One Health and global health security experts.*

Thank you for agreeing to take part in this interview. My name is ________ and I am a researcher from the London School of Hygiene and Tropical Medicine (LSHTM). I am part of a team are carrying out a study to develop a conceptual framework for assessing the sustainability of One Health initiatives. The first part of the interview focuses on how sustainability is conceptualized across human, animal and environmental sectors. The second part of the interview examines how the concept of sustainability is applied in a health security context by exploring its use within two widely used national capacity assessment tools – the World Health Organization Joint External Evaluation (JEE) and the World Organization for Animal Health Performance of Veterinary Services (PVS). The documents summarizing mentions of ‘sustainability’ in the two tools were shared with you through email already. I hope you have had a chance to look through these to help frame our discussion.

This interview will be audio-recorded and will take approximately 40-50 minutes.

Interviewee name:

Interviewee gender:

Interviewee nationality:

Interviewee geographical/country of origin:

Interviewee institutional affiliation/job title:

Interview start and end time:

**READ THROUGH THE INFORMED CONSENT FORM AND TAKE WRITTEN CONSENT BEFORE PROCEEDING.**

**A document review of the JEE and PVS tools was performed to identify reference to and measures of sustainability in these global assessment tools for health security. These were provided to you as a written summary prior to the interview.**

1. Do you think these sufficiently capture measures of sustainability in a health security capacity building context?
2. What is an appropriate timeframe for assessments of sustainability for your sector within a health security context?
3. From your perspective, what are the most important elements to sustain in health security strategies and how should we balance your sectoral interests and values with those of other One Health sectors?
4. Is there a need to ensure sustainability is built into national health security plans and strategies? Are there particular elements or determinants of sustainability that need to be explicitly measured or captured in tools like the JEE or PVS to embed the concept consistently in future health security planning?

Other notes or observations:

**AT THE END OF THE INTERVIEW REMEMBER TO ASK THE INTERVIEWEE IF THEY HAVE ANYTHING ELSE THEY WANT TO SAY, THANK THEM FOR TAKING PART, AND ASK IF THEY HAVE ANY QUESTIONS.**

**Appendix 4: COREQ checklist**

| **No. Item** | **Guide questions/description** | **Reported on Page #** |
| --- | --- | --- |
| **Domain 1: Research team and reﬂexivity** |  |  |
| *Personal Characteristics* |  |  |
| 1. Inter viewer/facilitator | Which author/s conducted the interview or focus group? | 1,7,8 |
| 2. Credentials | What were the researcher’s credentials? E.g. PhD, MD | 1,7,8 |
| 3. Occupation | What was their occupation at the time of the study? | 1,7,8 |
| 4. Gender | Was the researcher male or female? | N/A |
| 5. Experience and training | What experience or training did the researcher have? | 7,8 |
| *Relationship with participants* |  |  |
| 6. Relationship established | Was a relationship established prior to study commencement? | 7,8 |
| 7. Participant knowledge of the interviewer | What did the participants know about the researcher? e.g. personal goals, reasons for doing the research | 7,8 |
| 8. Interviewer characteristics | What characteristics were reported about the inter viewer/facilitator? e.g. Bias, assumptions, reasons and interests in the research topic | 8,9 |

| **Domain 2: study design** |  |  |
| --- | --- | --- |
| *Theoretical framework* |  |  |
| 9. Methodological orientation and Theory | What methodological orientation was stated to underpin the study? e.g. grounded theory, discourse analysis, ethnography, phenomenology, content analysis | 6,7 |
| *Participant selection* |  |  |
| 10. Sampling | How were participants selected? e.g. purposive, convenience, consecutive, snowball | 7,8 |
| 11. Method of approach | How were participants approached? e.g. face-to-face, telephone, mail, email | 7,8 |
| 12. Sample size | How many participants were in the study? | 7,8 |
| 13. Non-participation | How many people refused to participate or dropped out? Reasons? | 7,8 |
| *Setting* |  |  |
| 14. Setting of data collection | Where was the data collected? e.g. home, clinic, workplace | 7,8 |
| 15. Presence of non-participants | Was anyone else present besides the participants and researchers? | N/A |
| 16. Description of sample | What are the important characteristics of the sample? e.g. demographic data, date | 7,8 |
| *Data collection* |  |  |
| 17. Interview guide | Were questions, prompts, guides provided by the authors? Was it pilot tested? | 7,8 |
| 18. Repeat interviews | Were repeat inter views carried out? If yes, how many? | N/A |
| 19. Audio/visual recording | Did the research use audio or visual recording to collect the data? | 8,9 |
| 20. Field notes | Were ﬁeld notes made during and/or after the interview or focus group? | 8.9 |
| 21. Duration | What was the duration of the inter views or focus group? | 7,8 |
| 22. Data saturation | Was data saturation discussed? | 7,8 |
| 23. Transcripts returned | Were transcripts returned to participants for comment and/or correction? | N/A |
| **Domain 3: analysis and ﬁndings** |  |  |
| *Data analysis* |  |  |
| 24. Number of data coders | How many data coders coded the data? | 8,9 |
| 25. Description of the coding tree | Did authors provide a description of the coding tree? | 8,9 |
| 26. Derivation of themes | Were themes identiﬁed in advance or derived from the data? | 8,9 |
| 27. Software | What software, if applicable, was used to manage the data? | 8,9 |
| 28. Participant checking | Did participants provide feedback on the ﬁndings? | N/A |
| *Reporting* |  |  |
| 29. Quotations presented | Were participant quotations presented to illustrate the themes/ﬁndings? Was each quotation identiﬁed? e.g. participant number | 10-20 |
| 30. Data and ﬁndings consistent | Was there consistency between the data presented and the ﬁndings? | 10-20 |
| 31. Clarity of major themes | Were major themes clearly presented in the ﬁndings? | 10-20 |
| 32. Clarity of minor themes | Is there a description of diverse cases or discussion of minor themes? | 10-22 |
